# Improved prediction of sepsis-associated encephalopathy in intensive care unit sepsis patients with an innovative nomogram tool

**DOI:** 10.3389/fneur.2024.1344004

**Published:** 2024-02-20

**Authors:** Jun Jin, Lei Yu, Qingshan Zhou, Mian Zeng

**Affiliations:** ^1^Department of Intensive Care Unit, The University of Hong Kong-Shenzhen Hospital, Shenzhen, China; ^2^Department of Medical Intensive Care Unit, The First Affiliated Hospital, Sun Yat-sen University, Guangzhou, Guangdong, China; ^3^Institute of Pulmonary Diseases Sun Yat-sen University, Guangzhou, Guangdong, China

**Keywords:** sepsis, sepsis-associated encephalopathy, MIMIC-IV, nomogram, risk factor

## Abstract

**Background:**

Sepsis-associated encephalopathy (SAE) occurs as a result of systemic inflammation caused by sepsis. It has been observed that the majority of sepsis patients experience SAE while being treated in the intensive care unit (ICU), and a significant number of survivors continue suffering from cognitive impairment even after recovering from the illness. The objective of this study was to create a predictive nomogram that could be used to identify SAE risk factors in patients with ICU sepsis.

**Methods:**

We conducted a retrospective cohort study using the Medical Information Mart for Intensive Care IV (MIMIC-IV) database. We defined SAE as a Glasgow Coma Scale (GCS) score of 15 or less, or delirium. The patients were randomly divided into training and validation cohorts. We used least absolute shrinkage and selection operator (LASSO) regression modeling to optimize feature selection. Independent risk factors were determined through a multivariable logistic regression analysis, and a prediction model was built. The performance of the nomogram was evaluated using various metrics including the area under the receiver operating characteristic curve (AUC), calibration plots, Hosmer-Lemeshow test, decision curve analysis (DCA), net reclassification improvement (NRI), and integrated discrimination improvement (IDI).

**Results:**

Among the 4,476 sepsis patients screened, 2,781 (62.1%) developed SAE. In-hospital mortality was higher in the SAE group compared to the non-SAE group (9.5% vs. 3.7%, *p* < 0.001). Several variables were analyzed, including the patient’s age, gender, BMI on admission, mean arterial pressure, body temperature, platelet count, sodium level, and use of midazolam. These variables were used to create and validate a nomogram. The nomogram’s performance, assessed by AUC, NRI, IDI, and DCA, was found to be superior to the conventional SOFA score combined with delirium. Calibration plots and the Hosmer-Lemeshow test confirmed the accuracy of the nomogram. The enhanced NRI and IDI values demonstrated that our scoring system outperformed traditional diagnostic approaches. Additionally, the DCA curve indicated the practicality of the nomogram in clinical settings.

**Conclusion:**

This study successfully identified autonomous risk factors associated with the emergence of SAE in sepsis patients and utilized them to formulate a predictive model. The outcomes of this investigation have the potential to serve as a valuable clinical resource for the timely detection of SAE in patients.

## Introduction

Sepsis, a life-threatening infection-induced organ dysfunction resulting from a dysregulated host response (1), frequently affects the central nervous system (CNS), which is the most severely impacted and often overlooked organ system in sepsis (2). Research indicates that approximately 70% of sepsis patients develop SAE, a condition that can lead to prolonged mechanical ventilation, extended stays in the intensive care unit (ICU) and overall hospitalization period, and increased mortality (3). The pathophysiology of SAE involves the activation of microglial cells, neuroinflammation, and damage to the blood–brain barrier (4). Unfortunately, due to the lack of early diagnostic systems, the diagnosis and management of SAE are often delayed, resulting in increased rates of morbidity and mortality. Therefore, early identification and treatment of SAE are critical for the survival and prognosis of sepsis patients (5).

There is limited data available for predicting the occurrence of SAE in sepsis patients. In clinical practice, the presence of sepsis-associated delirium is often utilized as a substitute for diagnosing SAE (6), and delirium is evaluated by using a subjective scoring method known as CAM-ICU. Despite the existence of some biomarkers like neurofilament light chains (NFL), S100 calcium-binding protein B (S100β), and neuron-specific enolase (NSE) for predicting the occurrence of SAE (7–9), these biomarkers are not readily accessible in clinical settings and lack the necessary sensitivity and specificity for accurately predicting SAE. Consequently, new predictive indicators, including clinical parameters, are required to aid in prediction. The objective of this study is to retrospectively analyze sepsis-related data obtained from a large public database to establish a predictive model that incorporates potential risk factors for early identification of SAE.

## Materials and methods

### Data source

The MIMIC-IV (Medical Information Mart for Intensive Care-IV) database is an openly accessible repository of medical information pertaining to intensive care. Specifically, the MIMIC-IV 2.2 version, as of 6 January 2023, encompasses authentic data collected from the ICU of the Massachusetts Institute of Technology Beth Israel Deaconess Medical Center during the period spanning from 2008 to 2019 (10). Authors have obtained permission to use the database (Certificate No.:38078934).

### Study population

**Figure 1:**
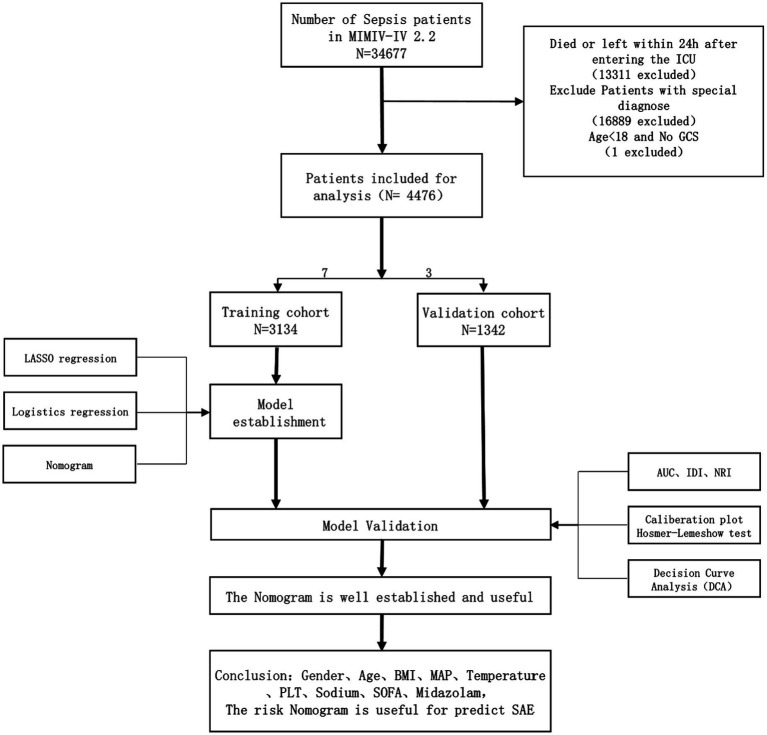
Workflow of the study. GCS Glasgow Coma Scale, SOFA Sequential Organ Failure Assessment. BMI, Body Mass Index; MAP, Mean Arterial Pressure; PLT, Platelet; AUC, Area Under the Curve; IDI, Integrated Discrimination Improvement; NRI, Net Reclassification Improvement. Special diagnostics include primary neurological injuries (traumatic brain injuries, ischemic strokes, hemorrhagic strokes, epilepsy, and intracranial infections), and chronic alcohol or drug abuse, severe electrolyte imbalances, hyponatremia, hyperglycemia, hypoglycemia, with pre-existing liver or kidney failure affecting consciousness, receiving cardiac resuscitation recently.

In this investigation, SAE is defined as a Glasgow Coma Scale (GCS) score lower than 15 or a diagnosis of delirium (11). The Glasgow Coma Scale (GCS) was first described in 1974 and has since been widely used to assess a patient’s level of consciousness upon admission. GCS evaluates a person’s level of consciousness based on three aspects: eye response, verbal response, and motor response. The GCS score ranges from 3 to 15 points and is related to the level of consciousness. The GCS is a quick, widely available, and standardized tool for assessing consciousness and neurological function. A GCS score less than 15 indicates some degree of impaired consciousness, which can be a sign of brain dysfunction. Delirium is included as a criterion, even in patients with a GCS of 15, because it represents a change in mental status with a fluctuating course, inattention, and either disorganized thinking or an altered level of consciousness. Delirium is particularly relevant as it is a common manifestation of septic encephalopathy and can occur even when the level of consciousness seems intact (GCS 15). Our study excluded consciousness disorders with a clear etiology. To be included, subjects needed to meet the following criteria: (1) be above the age of 18; (2) fulfill the diagnostic criteria for Sepsis 3.0; (3) have a hospital stay exceeding 24 h; (4) be admitted to the Intensive Care Unit for the first time; (5) have their initial patient information recorded. Conversely, individuals were excluded if they met any of the following exclusion criteria: (1) previously diagnosed with brain injuries (such as traumatic brain injury, meningitis, encephalitis, cerebral hemorrhage, cerebral embolism, ischemic stroke, epilepsy, brain tumor, intracranial infection, or any other cerebrovascular disease); (2) had mental disorders or neurological diseases; (3) suffered from long-term alcoholism or drug abuse; (4) experienced metabolic encephalopathy, hepatic encephalopathy, hypertensive encephalopathy, or any other liver or kidney disease affecting consciousness; (5) suffered from severe electrolyte imbalance or blood glucose disorder, including hyponatremia (less than 120 mmoL/L), hyperglycemia (greater than 180 mg/dL), or hypoglycemia (less than 54 mg/dL); (6) have not been evaluated using the GCS. The detailed process of data inclusion can be observed in Figure 1.

### Study methods

Using the software Navicat and employing Structured Query Language (SQL) (12), we acquired a total of 43 variables. These variables encompassed various domains, including: (1) Baseline data: age, sex, race, BMI, and comorbidities. (2) Vital signs: GCS score, mean arterial pressure, heart rate, respiratory rate, pulse oximetry (SpO2), and body temperature. (3) Laboratory tests: hematocrit, hemoglobin, red cell distribution width, platelets, white blood cells, prothrombin time, partial thromboplastin time, blood urea nitrogen, electrolytes, and blood gas analysis. (4) Type of microbial infection. (5) Comorbidities and disease severity scores are evaluated using the Charlson comorbidity index and the Sequential Organ Failure Assessment (SOFA) score. The Charlson comorbidity index measures the presence and impact of various comorbid conditions, while the SOFA score assesses the degree of organ dysfunction or failure across six specific organ systems: coagulation, liver, kidney, cardiovascular, lung, and neurological. Each organ system is rated on a scale from 0 (indicating normal function) to 4 (indicating severe abnormalities). (6) Administration of sedative drugs: midazolam and propofol. (7) Utilization of mechanical ventilation and indication of post-elective surgeryThe primary focus of this investigation was the prevalence of SAE. For continuous variables, we calculated the average values from both the laboratory parameters and vital sign parameters over the initial 24-h period. All categorical variables underwent preprocessing before being introduced into the model.

### Statistical methods

The research subjects were divided into two groups based on the incidence of SAE. Variables that had more than 30% missing values were not included in the analysis (13). For variables with less than 30% missing values, multiple imputations were performed. The procedure starts by predicting and filling in missing values for the first variable using a regression model. Then, the imputed values for the first variable are kept constant, and the process is repeated for the second variable. This cycle is continued for all variables, with multiple iterations to improve the accuracy of the estimates. We perform 5 iterations, resulting in 5 complete data sets. The final analysis is based on the average of these 5 data sets to account for the uncertainty in the estimates. The data distribution was analyzed using the Shapiro–Wilk test. Continuous data was represented as mean ± standard deviation or median (interquartile range, IQR), while categorical variables were presented as frequencies and ratios (%). Non-parametric tests (Mann–Whitney U test or Kruskal-Wallis test) were employed for non-normally distributed or heteroscedastic data. Pearson’s chi-square test was used to compare categorical data. All statistical analyses were carried out using R software, utilizing various packages including tableone, mice, rms, pROC, dca, and rdma.

To develop the prediction nomogram (a graphical tool for predicting disease probability based on multiple predictive factors. The user plots points for each factor on a scale, sums these points, and then translates the total into a probability of disease occurrence). A random assignment of patients was made into a training set and a validation set, with a ratio of 7:3. The variable selection was performed using the Lasso regression method, also known as the least absolute shrinkage and selection operator. LASSO is a modeling technique that selects variables by constraining the sum of regression coefficients, ultimately choosing variables for the model and penalizing fewer predictive variables to prevent overfitting. The optimal value of λ was determined through 10-fold cross-validation. Subsequently, a multivariable logistic regression analysis was conducted using the features selected from the LASSO regression model to identify statistically significant predictors. These predictors were then used to construct a nomogram. The results of this analysis are presented as odds ratios (OR), 95% confidence intervals (95% CI), and *p*-values.

The discriminative ability of the nomogram and the SOFA delirium system was evaluated by assessing the area under the receiver operating characteristic curve (AUC). The receiver operating characteristic curve was used to determine the optimal cut-off point, as well as the sensitivity and specificity based on the Youden index. Furthermore, the performance improvement of the nomogram compared to the SOFA plus delirium system was assessed using the Integrated Discrimination Improvement (IDI) and the Net Reclassification Improvement (NRI). Both are statistical measures used to compare the performance of two predictive models. IDI focuses on the improvement in discrimination, essentially how well the new model differentiates between outcomes compared to the old model. NRI assesses how well individuals are reclassified into correct risk categories by the new model vs. the old one. Together, they help in understanding whether a new model offers a significant improvement over its predecessor. Calibration curves and the Hosmer-Lemeshow test were utilized to evaluate the calibration of the nomogram. The net clinical benefit was determined through the decision curve analysis (DCA) curve which a method used to evaluate the clinical utility of diagnostic tests or prediction models across a range of decision thresholds. It helps in understanding the trade-offs between the benefits of true positive results and the harms of false positives. DCA is particularly useful for determining at what threshold a model’s predictions have practical value, guiding clinicians on when to use the model for decision-making.

All statistical analyses were conducted using R software version 4.3.1 (R Foundation for Statistical Computing, Vienna, Austria). Significance was defined as a *p*-value less than 0.05 (two-sided).

## Results

Currently, there is a lack of well-defined diagnostic criteria for SAE, resulting in its frequent substitution with the diagnosis of delirium in conjunction with sepsis (SOFA + Delirium). The present study aimed to develop a diagnostic prediction model for SAE by gathering data from the MIMIC-IV database.

### Baseline information and clinical data

The 4,476 patients who met inclusion and exclusion criteria were randomly divided into a training set of 3,134 and a validation set of 1,342 with a 7:3 ratio. The demographic, clinical, and laboratory characteristics of the two groups of patients are shown in Table 1. There was no statistical difference in all observed variables between training group and validation group (*p* > 0.05).

**Table 1:** Baseline characteristics of study participants with and without SAE.

Variables	Total (*N* = 4,476)	SAE (*N* = 2,781)	Non-SAE (*N* = 1,695)	*p*
Age (IQR)	67.00(56.00,76.00)	68.00(56.00, 77.00)	65.00(55.00, 73.00)	<0.001
BMI (IQR)	27.76(24.24,32.25)	27.53(24.05, 31.96)	28.08(24.63, 32.67)	<0.001
Gender, *n* (%)				<0.001
Male	2,722 (60.8%)	1,611 (57.9%)	1,111 (65.5%)	
Female	1,754 (39.2%)	1,170 (42.1%)	584 (34.5%)	
Race, *n* (%)				0.597
White	3,172 (70.9%)	1,982 (71.3%)	1,190 (70.2%)	
Black	225 (5.0%)	146 (5.2%)	79 (4.7%)	
Asian	130 (2.9%)	75 (2.7%)	55 (3.2%)	
Hispanic/Latino	130 (2.9%)	77 (2.8%)	53 (3.1%)	
Others	819 (18.3%)	501 (18.0%)	318 (18.8%)	
**Comorbidities**				
Hypertension, *n* (%)				0.645
No	1,642 (36.7%)	1,013 (36.4%)	629 (37.1%)	
Yes	2,834 (63.3%)	1,768 (63.6%)	1,066 (62.9%)	
Congestive heart failure, *n* (%)				0.637
No	3,631 (81.1%)	2,250 (80.9%)	1,381 (81.5%)	
Yes	845 (18.9%)	531 (19.1%)	314 (18.5%)	
Chronic pulmonary disease, *n* (%)				0.862
No	3,506 (78.3%)	2,176 (78.2%)	1,330 (78.5%)	
Yes	970 (21.7%)	605 (21.8%)	365 (21.5%)	
Diabetes Mellitus, *n* (%)				0.987
No	4,236 (94.6%)	2,632 (94.6%)	1,604 (94.6%)	
Yes	240 (5.4%)	149 (5.4%)	91 (5.4%)	
Cancer, *n* (%)				<0.001
No	3,917 (87.5%)	2,394 (86.1%)	1,523 (89.9%)	
Yes	559 (12.5%)	387 (13.9%)	172 (10.1%)	
**Scoring systems (IQR)**				
Sofa score	5.00(3.00,7.00)	6.00(4.00, 8.00)	4.00(3.00, 5.00)	<0.001
Charlson comorbidity index,	4.00(3.00,5.00)	4.00(3.00, 6.00)	4.00(3.00, 5.00)	<0.001
GCS	14.00(11.00,15.00)	13.00(8.00, 14.00)	15.00(15.00, 15.00)	<0.001
**First day vital signs (IQR)**				
Heart rate mean (beats min^−1^)	84.03(76.73,93.96)	84.50(76.68, 95.52)	83.39(77.02, 92.26)	0.008
MAP mean (mmHg)	74.86(70.67,79.72)	75.11(70.57, 80.23)	74.60(70.82, 78.85)	0.061
Respiratory Rate mean (min^−1^)	18.00(16.14,20.45)	18.11(16.15, 20.76)	17.83(16.13, 19.90)	0.001
Temperature mean (°C)	36.84(36.59,37.17)	36.86(36.60, 37.20)	36.80(36.57, 37.10)	<0.001
Spo2 mean (%)	97.70(96.41,98.70)	97.70(96.37, 98.70)	97.71(96.50, 98.69)	0.587
First day laboratory tests (IQR)	84.03(76.73,93.96)	84.50(76.68, 95.52)	83.39(77.02, 92.26)	0.008
Hematocrit (%)	31.30(28.25,34.86)	31.20(28.05, 34.90)	31.40(28.40, 34.80)	0.218
Hemoglobin (g/dL)	10.50(9.45,11.75)	10.50(9.40, 11.70)	10.55(9.55, 11.80)	0.095
Platelet (10^3^/uL)	169.50(132.50,224.50)	175.00(133.50, 233.00)	163.00(131.00, 211.00)	<0.001
WBC (10^3^/uL)	12.45(9.65,15.85)	12.40(9.65, 15.80)	12.55(9.70, 15.90)	0.465
RDW (%)	14.00(13.20,15.30)	14.10(13.30, 15.50)	13.80(13.20, 14.80)	<0.001
Cr mean (mg/dL)	0.85(0.70,1.05)	0.85(0.70,1.05)	0.85(0.70,1.00)	0.015
BUN mean (mg/dL)	15.50(12.50,21.00)	16.00(12.50, 22.00)	15.50(12.00, 19.50)	<0.001
Glucose mean (mg/dL)	119.00(105.00,134.00)	119.50(105.50, 135.00)	119.00(105.00, 132.50)	0.117
Sodium mean (mmol/L)	138.50(136.50,140.50)	138.50(136.50, 140.50)	138.50(136.50, 140.00)	0.002
PT mean (S)	14.15(13.10,15.45)	14.25(13.10, 15.60)	14.10(13.10, 15.25)	0.007
PTT mean (S)	30.80(27.60,36.60)	30.85(27.55, 37.05)	30.80(27.67, 35.95)	0.499
Lactate mean (mmol/L)	1.80(1.35,2.35)	1.75(1.35, 2.40)	1.80(1.40, 2.30)	0.194
pH mean	7.38(7.35,7.42)	7.38(7.35, 7.42)	7.38(7.35, 7.41)	0.132
PO2 mean (mmHg)	227.25(126.00,272.00)	217.50(116.50, 268.00)	240.50(153.50, 277.00)	<0.001
PCO2 mean (mmHg)	41.00(37.50,44.50)	40.50(37.00, 44.50)	41.00(38.00, 44.50)	0.012
Phosphate (mg/dL)	3.40(2.90,4.00)	3.40(2.80, 4.00)	3.40(2.90, 3.90)	0.394
Magnesium (mg/dL)	2.00(1.80,2.20)	2.00(1.80, 2.20)	2.00(1.80, 2.20)	<0.001
**Interventions, *n* (%)**				
Midazolam, *n* (%)				<0.001
No	3,542 (79.1%)	2,060 (74.1%)	1,482 (87.4%)	
Yes	934 (20.9%)	721 (25.9%)	213 (12.6%)	
Propofol, *n* (%)				0.007
No	906 (20.2%)	598 (21.5%)	308 (18.2%)	
Yes	3,570 (79.8%)	2,183 (78.5%)	1,387 (81.8%)	
Mechanical ventilation, *n* (%)				0.771
No	1,984 (44.3%)	1,228 (44.2%)	756 (44.6%)	
Yes	2,492 (55.7%)	1,553 (55.8%)	939 (55.4%)	
Elective surgery, *n* (%)				<0.001
No	3,981 (88.9%)	2,507 (90.1%)	1,474 (87.0%)	
Yes	495 (11.1%)	274 (9.9%)	221 (13.0%)	
Vasopressor, *n* (%)				0.077
No	1,559 (34.8%)	996 (35.8%)	563 (33.2%)	
Yes	2,917 (65.2%)	1,785 (64.2%)	1,132 (66.8%)	
Delirium, *n* (%)				<0.001
No	4,290 (95.8%)	2,595 (93.3%)	1,695 (100.0%)	
Yes	186 (4.2%)	186 (6.7%)	0 (0.0%)	
Microorganism, *n* (%)				0.007
Negative	3,845 (85.9%)	2,378 (85.5%)	1,467 (86.5%)	
Gram Negative	221 (4.9%)	159 (5.7%)	62 (3.7%)	
Gram positive	385 (8.6%)	226 (8.1%)	159 (9.4%)	
Others	25 (0.6%)	18 (0.6%)	7 (0.4%)	
**Outcome-related measures**				
Length of hospital (days)	7.13(5.07,11.42)	8.08(5.29, 13.57)	6.13(4.77, 8.96)	<0.001
Length of ICU (days)	2.29(1.34,4.29)	2.99(1.50, 5.89)	1.83(1.24, 2.86)	<0.001
Hospital mortality, *n* (%)				<0.001
No	4,149 (92.7%)	2,517 (90.5%)	1,632 (96.3%)	
Yes	327 (7.3%)	264 (9.5%)	63 (3.7%)	

BMI, Body Mass Index; SOFA, Sequential Organ Failure Assessment; GCS, Glasgow Coma Scale; MAP, Mean Arterial Pressure; SpO2, Pulse Oximetry; PLT, Platelet; WBC, White Blood Cell; RDW, Red Blood Cell Distribution Width; BUN, Blood Urea Nitrogen; PT, Prothrombin Time; PTT, Partial Thromboplastin Time.

### Results of LASSO regression screening

**Figure 2:**
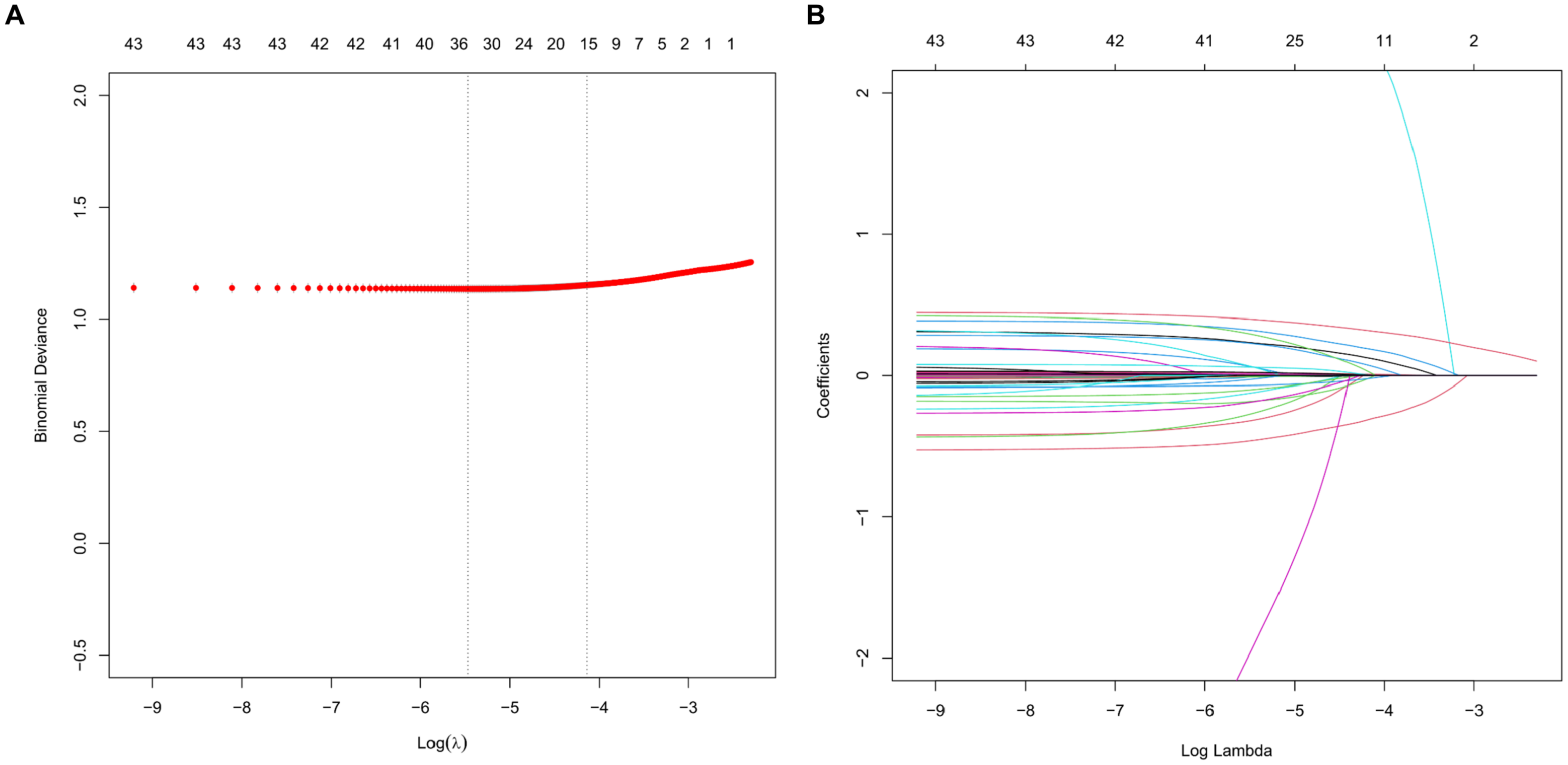
LASSO regression analysis and 10-fold cross-validation were used to select predictor variables. **(A)** The adjustment parameter (lambda) for LASSO regression deviance is selected based on the minimum criterion (dashed line on the left) and the 1-SE criterion (dashed line on the right). **(B)** Create coefficient distributions from log(lambda) sequences. In the present study, predictor’s selection was according to the 1-SE criteria (dashed line on the right), where 9 nonzero coefficients were selected. LASSO, least absolute shrinkage and selection operator; SE, standard error.

The study utilized the LASSO regression algorithm to identify the most significant predictive factors in order to avoid overfitting. The selection of the best parameter (Lambda) in the LASSO model was determined through 10-fold cross-validation using the smallest criteria. The optimal value for the model was determined to be one standard error of the smallest Lambda value (Lambda1se). The results of the LASSO analysis revealed that several factors, including average gender, age, BMI, congestive heart failure, MAP, temperature, SpO2, PLT, sodium, lactate, pH value, use of midazolam, use of vasoactive drugs, type of microorganism, and SOFA score, were identified as risk factors for the occurrence of SAE (Figure 2).

### A multivariate logistic regression of risk factors

**Figure 3:**
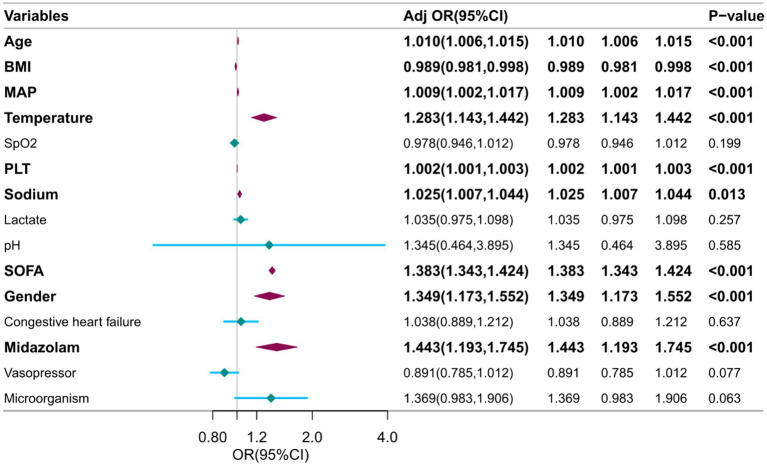
Multivariable logistic regression to identify optimal predictors for SAE diagnosis. BMI, Body Mass Index. SOFA, Sequential Organ Failure Assessment. MAP, Mean Arterial Pressure. PLT, Platelet.

The independent variables chosen through LASSO regression are utilized in multivariate logistic regression analysis, with SAE serving as the dependent variable. Following the elimination of variables with a *p*-value exceeding 0.05, a total of nine factors are found to be significantly associated with SAE, namely gender, age, BMI, MAP, temperature, platelet count, sodium levels, use of midazolam, and SOFA score (Figure 3).

### Construction and evaluation of a nomogram

**Figure 4:**
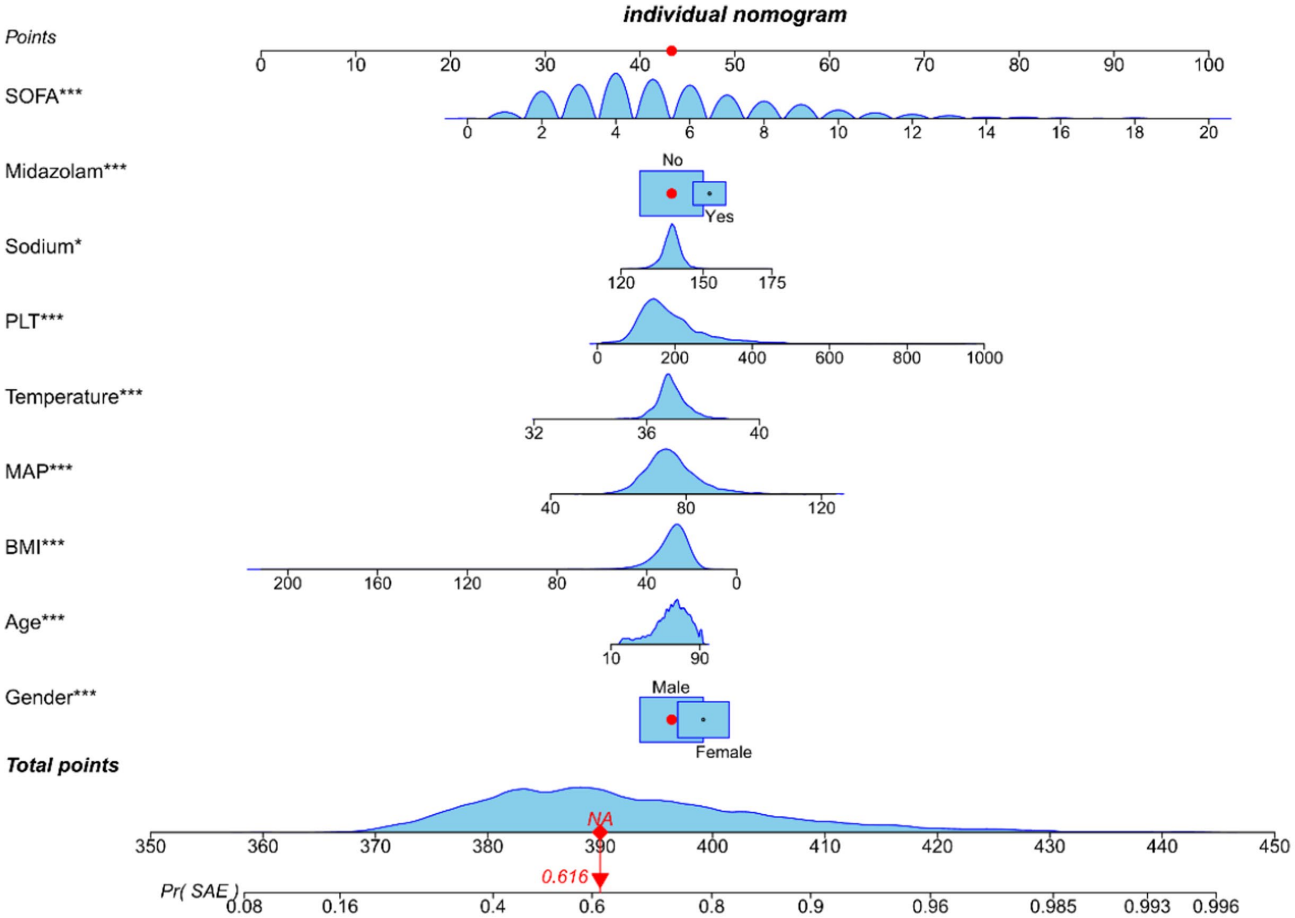
Nomogram predicting SAE probability in sepsis patients of training cohort. In order to obtain the corresponding scores for each variable, draw a vertical line upward from the point axis. The total score at the bottom of the nomogram represents the probability of SAE based on the sum of all variable scores. Those red dots represent symptoms and probabilities of SAE in our study population.

Utilizing the nine variables derived from the multivariate regression analysis as predictors, with the occurrence of SAE as the clinical outcome, a nomogram was constructed (Figure 4).

**Figure 5:**
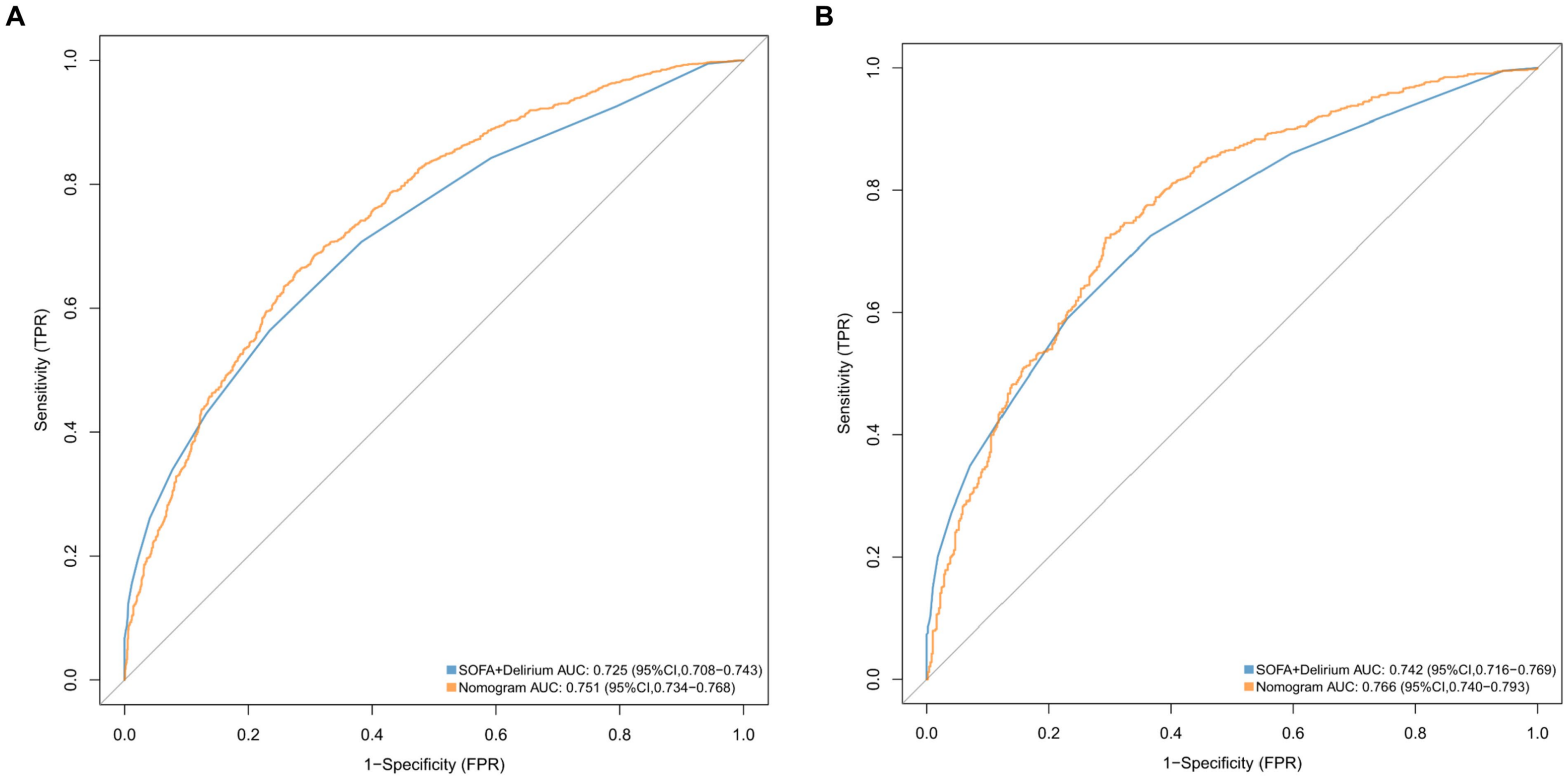
ROC curves for the nomogram model and the SOFA + Delirium model in validation cohort **(A)** and the training cohort **(B)**.

We conducted a comparison of the diagnostic predictive ability between the nomogram and the combination of the SOFA score and the delirium system for SAE. The results, as shown in Figure 5, indicate that the nomogram had higher AUC values in both the training set (0.751, 95% CI = 0.734–0.768; Figure 5A) and the validation set (0.766, 95% CI = 0.740–0.793; Figure 5B) compared to the SOFA score combined with the delirium diagnostic system. Furthermore, the NRI values of categorical variables in the training set were 0.0493 (95% CI = 0.0171–0.0808), while the NRI values of continuous variables were 0.3484 (95% CI = 0.278–0.4189), and the IDI value was 0.0279 (95% CI = 0.0194–0.0364). In the validation set, the NRI values of categorical variables and continuous variables were 0.0504 (95% CI = 0.0042–0.0965) and 0.3814 (95% CI = 0.2731–0.489), respectively, with a corresponding IDI value of 0.0268 (95% CI = 0.0139–0.0397; Table 2). These findings suggest that our model improves prediction accuracy and outperforms the currently utilized diagnostic tools for SAE.

**Table 2:** Comparison of the performance of two models for predicting SAE.

Predict model		AUROC	*P*-value	NRI (categorical)	*P-*value	NRI (continuous)	*P*-value	IDI	*P*-value
Training set	Nomogram	0.751							
	SOFA + Delirium	0.725	<0.001	0.0493[0.0177–0.0808]	0.002	0.3484[0.278–0.4189]	<0.001	0.0279[0.0194–0.0364]	<0.001
Validation set	Nomogram	0.766							
	SOFA + Delirium	0.742	0.008	0.0504[0.0042–0.0965]	0.03	0.3814[0.2731–0.4897]	<0.001	0.0268[0.0139–0.0397]	<0.001

The *P*-value was calculated by comparing the results of nomogram with SOFA plus Delirium. SOFA, Sequential Organ Failure Assessment; AUROC, Area Under the ROC Curve; NRI, Net Reclassification Improvement; IDI, Integrated Discrimination Improvement.

**Figure 6:**
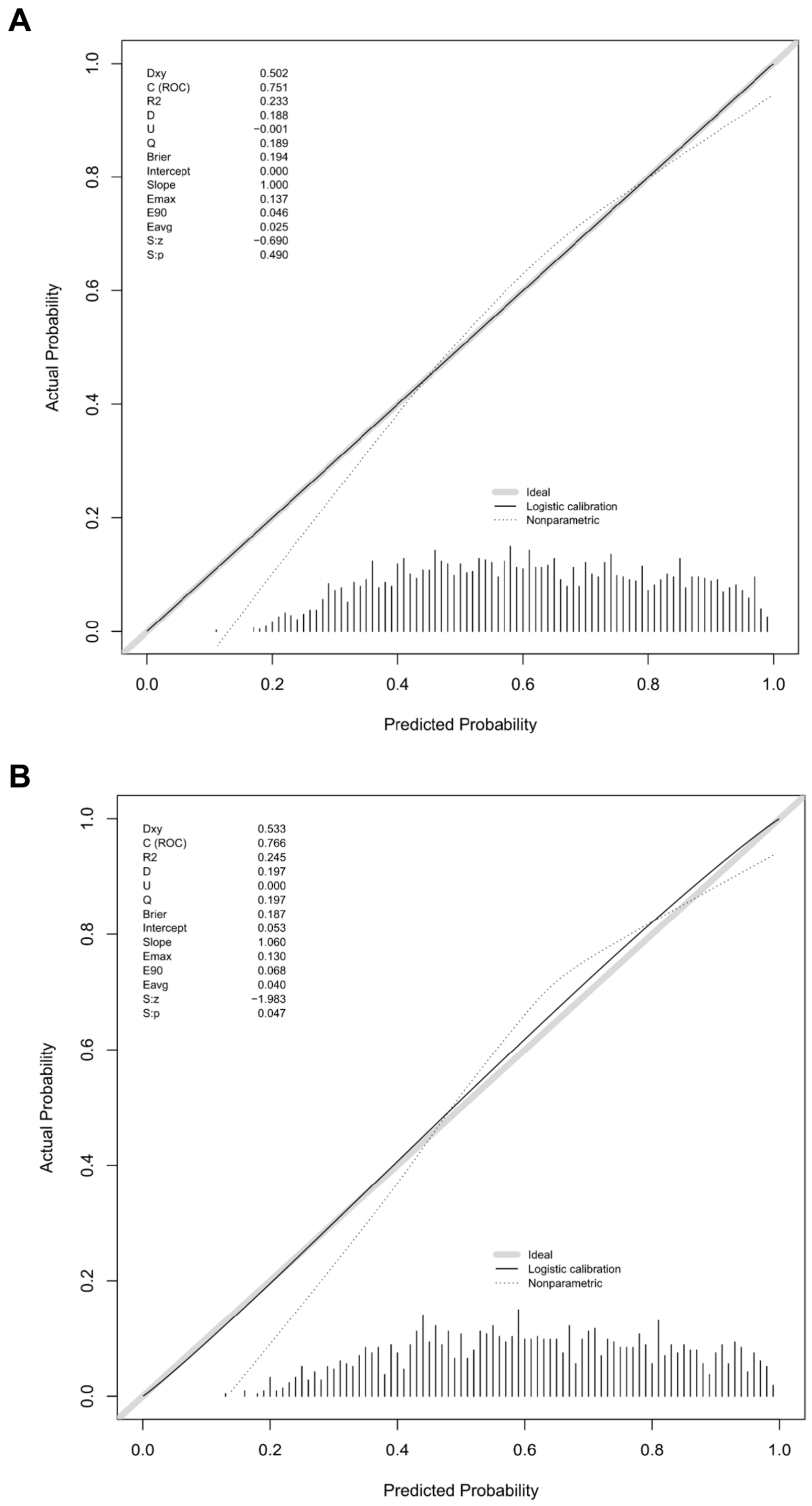
Calibration curves for the validation cohort **(A)** and the training cohort **(B)**.

**Figure 7:**
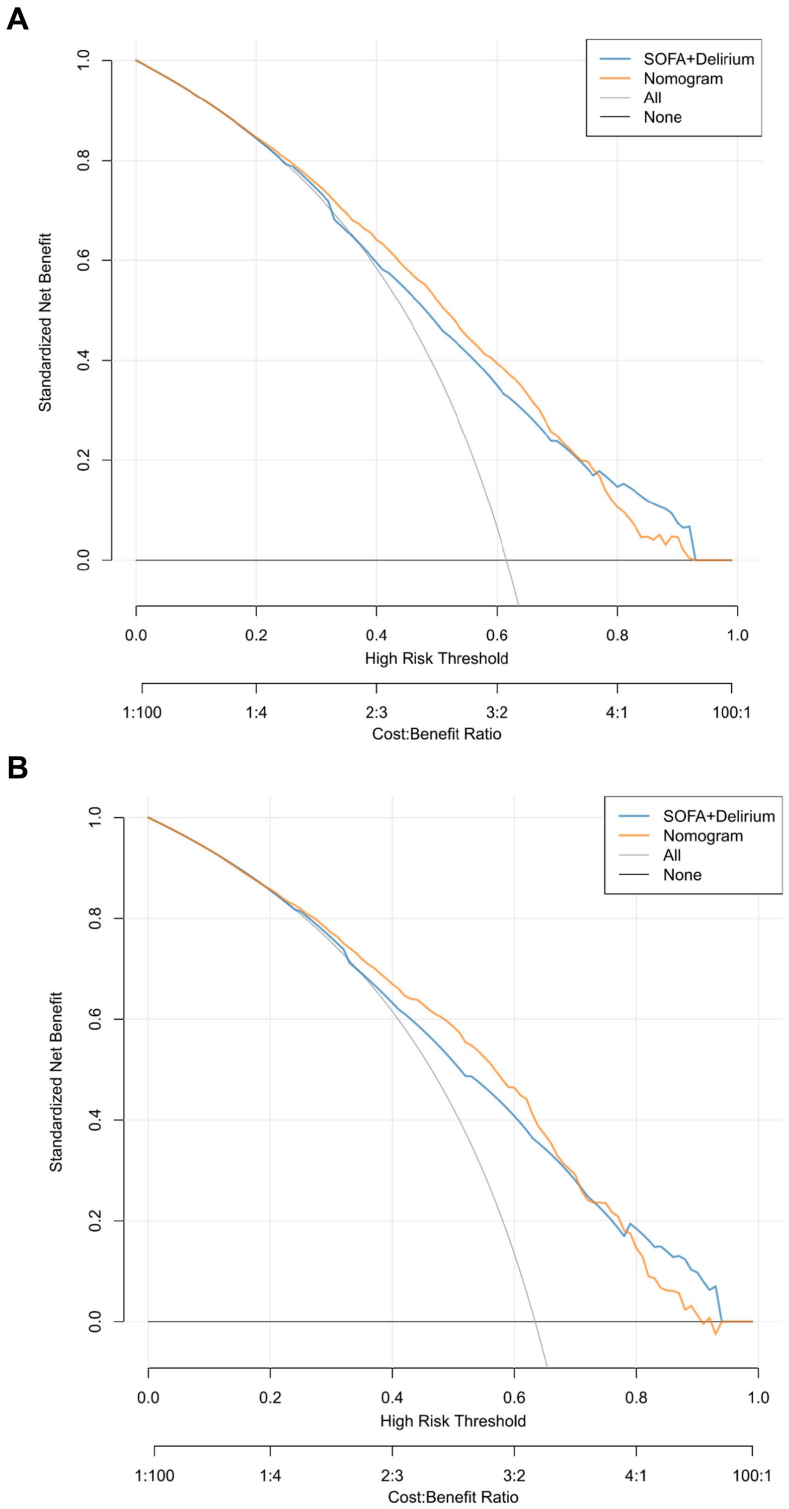
Decision-curve analysis of the validation cohort **(A)** and the training cohort **(B)**.

Figure 6 presents the calibration curve of the nomogram. The calibration curves for both the training (Figure 6A) and validation (Figure 6B) cohorts are nearly diagonal. The results of the Hosmer-Lemeshow test indicate no statistical significance, suggesting a good fit between the nomogram and the data (training cohort: Χ^2^ = 12.598, *p* = 0.1264; validation cohort: Χ^2^ = 8.6418, *p* = 0.3734). To demonstrate the clinical applicability of the nomogram, we also plotted the DCA curve and compared it with the combination of the SOFA score and the delirium diagnostic system (Figure 7). Our nomogram showed a higher net benefit in clinical diagnosis when the threshold probabilities ranged from 0.13 to 0.92 (Figure 7A) and 0.19 to 0.9 (Figure 7B) for the two cohorts, respectively, surpassing the currently used scoring system.

## Discussion

In this retrospective study using the MIMIC-IV database, the incidence of SAE was found to be 62.1%. We identified several independent risk factors for SAE, including gender, age, BMI, mean arterial pressure, temperature, platelets, sodium, use of midazolam, and SOFA score. Based on these findings, we developed a diagnostic prediction nomogram for SAE. The validity of our nomogram model was evaluated using multiple indicators such as AUC, calibration curve, Hosmer-Lemeshow test, IDI, NRI, and DCA, which demonstrated high validity, discrimination, and clinical utility.

Infections with pathogenic pathogens can result in disturbance of the immune response in the host, ultimately leading to severe dysfunction of organs (14). The existing definition of sepsis, referred to as the “Sepsis-3” criterion, emphasizes the occurrence of organ failure in sepsis patients and necessitates the evaluation of sequential organ failure with a minimum score of two (1). It is estimated that sepsis affected approximately 49 million individuals globally in 2017, resulting in the deaths of 11 million people (15). We have also observed a gradual reduction in the mortality rate of sepsis adjusted for age, which could be attributed to advancements in clinical guidelines and care. These improvements have consequently increased the number of sepsis patients who survive the condition (16). In terms of acute brain dysfunction resulting from sepsis, approximately 50% of sepsis patients admitted to the intensive care unit (ICU) exhibited symptoms such as delirium and coma. This neurological manifestation, unrelated to direct brain infection by the pathogen, is recognized as SAE (17). Various research studies have extensively linked SAE to higher short-term mortality among sepsis patients (5, 18, 19). Furthermore, our investigation discovered a notably increased in-hospital mortality rate for patients in the SAE group, as well as an extended duration of ICU stay (*p* < 0.001). Currently, the diagnostic criteria and potential risk factors for SAE remain inadequately understood, and there is an absence of reliable methods for the clinical assessment of sepsis-induced neurological dysfunction. Consequently, the development of an early diagnostic predictive model could aid in the diagnosis of SAE and facilitate treatment decision-making.

Although sepsis can develop in patients of any age, age is a powerful risk factor, with patients over 65 experiencing a more than tenfold increase in incidence compared to younger individuals (18–49 years). The majority of sepsis survivors (56%) are over the age of 65. Among this group, half do not fully recover and instead experience new functional impairments. These impairments are a result of the decline in physiological reserves and immune function, which aligns with the findings of our study (14, 20). We also found that female patients were more likely to develop SAE, considering differences in the immune system, brain tissue structure, blood–brain barrier, and neuroendocrine system between males and females (21). However, this contradicts the findings of Feng’s study (22). Many current studies on SAE have matched for age and gender, indicating an understanding of the significant impact of these two factors on disease progression. However, data elucidating the exact way gender influences SAE are lacking, indicating an urgent need for further research. Although research suggests that a higher BMI in middle age is associated with dementia, the same studies have also found that a higher BMI in old age may be a protective factor (23). Furthermore, a low BMI is associated with more severe neurodegenerative diseases and a higher mortality rate (24). We also found that patients with a low BMI were more likely to develop SAE, as low BMI often indicates malnutrition, which can affect brain function and cause gut-brain axis dysfunction, leading to SAE.

Additionally, our study found that mean arterial pressure (MAP) and body temperature among vital signs were associated with the occurrence of SAE. MAP is the pressure that most significantly affects autoregulation of blood flow within organs. A MAP of 65-70 mmHg is the initial systemic circulation target to ensure organ perfusion pressure (25). However, patients with sepsis often have impaired cerebrovascular autoregulation (26). Schramm et al. confirmed through TCD that cerebrovascular autoregulation dysfunction is one of the triggers for SAE (27). We know that low MAP can lead to insufficient cerebral blood flow and cerebral perfusion disorders; conversely, if MAP is too high and exceeds the range of cerebrovascular autoregulation, it can also lead to increased intracranial pressure and a decrease in cerebral perfusion pressure, which is consistent with our research results. Therefore, individualized MAP based on cerebrovascular autoregulation monitoring is needed to prevent SAE (28). Sepsis itself is a systemic inflammatory state caused by severe infection. The inflammatory response can disrupt thermoregulatory mechanisms, leading to abnormalities such as fever or hypothermia, and prolonged high fever can exacerbate blood–brain barrier damage and the degree of neuronal necrosis during SAE (29, 30).

Thrombocytopenia is a common complication of sepsis, and previous studies have confirmed that thrombocytopenia is associated with poor prognosis in patients with SAE (30). However, our study suggests that thrombocytosis upon admission is associated with the occurrence of SAE. This is primarily because platelets can release cytokines and neurotransmitters, such as serotonin, IL-1β, and platelet activating factor, which can promote inflammatory responses and leukocyte migration (31). Additionally, platelet surface receptors, such as GPIb, can bind to leukocyte surface receptors, such as Mac-1, promoting leukocyte adhesion to the vascular endothelium and entry into brain tissue, thereby triggering neuroinflammation (32). Sodium, an essential electrolyte for nerve cells and a significant component of plasma osmotic pressure, can cause an increase in plasma osmotic pressure in hypernatremia, leading to a transfer of water from the brain to nerve cells. This can result in symptoms such as somnolence, epilepsy, and delirium (33, 34). Our study found that hypernatremia is also an independent risk factor for SAE, consistent with the findings of Romain Sonneville’s study (30).

Midazolam, a benzodiazepine drug, is commonly used for sedation in ICU patients. However, it may increase the risk of neurocognitive impairment by affecting β-amyloid protein clearance, increasing tau protein levels, exacerbating the inflammatory response, and affecting synaptic plasticity. Previous studies have shown that the use of midazolam is an independent risk factor for predicting the development of neurocognitive impairment in ICU patients, increasing their risk (35, 36). Our study also confirms that the use of midazolam is one of the risk factors for SAE.

In terms of sepsis diagnosis, the most commonly used tool is the SOFA score, which is also one of the diagnostic criteria for sepsis 3.0. Previous studies have reported that the SOFA score has good diagnostic and prognostic predictive value in sepsis patients (37). However, whether it is applicable for the diagnosis of SAE is currently unclear (38). Therefore, we extracted data from the MIMIC-IV dataset to develop the current prediction model. The results show that our prediction model is superior to the current SOFA combined with delirium diagnostic system and displays acceptable discrimination and calibration. In addition, guided by the nomogram, we performed a clinical decision analysis for the diagnosis of SAE patients and found that the current prediction model has a higher net benefit. Note that a GCS score of less than 15 is used to diagnose SAE patients; however, the GCS score is part of the SOFA score. Patients with a higher SOFA score are more likely to develop SAE, which may bias this conclusion.

Based on our current knowledge, there exists a dearth of research pertaining to the predictive model for diagnosing SAE. In our investigation, we employed the publicly accessible MIMIC-IV database, which encompasses an extensive array of data from critically ill individuals. This database served as a reliable source of evidence for our discoveries. The parameters integrated into the SAE prediction model developed in this study can be readily obtained through clinical practices. Furthermore, the model possesses interpretability, thereby conferring significant value for the early prognosis of SAE within clinical environments.

However, our study has some limitations. Firstly, the absence of clearly defined diagnostic criteria for SAE poses a challenge. Although we established some inclusion and exclusion criteria, misdiagnoses and missed diagnoses are bound to occur. Secondly, our study extensively relied on the MIMIC-IV database, which is known for its homogeneity. Consequently, we only conducted internal validation with this specific database. To enhance the model’s robustness and performance, the inclusion of external databases in future investigations is imperative. Moreover, it is important to acknowledge that our study is retrospective by nature, which inherently introduces biases. Lastly, due to limitations in data availability, several laboratory tests, including PCT, CRP, and IL-6, were not feasible to obtain. Consequently, these potential risk factors could not be included in the prediction model.

## Conclusion

A diagnostic tool was developed and evaluated for its precision and discriminative efficacy in diagnosing SAE. This novel nomogram provides healthcare professionals with a personalized and visual tool that may aid in timely intervention and reduce mortality associated with SAE. To evaluate the predictive performance of the model across different populations, prospective studies with external validation are imperative.

## Data availability statement

The data analyzed in this study was obtained from the Medical Information Mart for Intensive Care IV (MIMIC-IV) database, the following licenses/restrictions apply: to access the files, users must be credentialed users, complete the required training (CITI Data or Specimens Only Research), and sign the data use agreement for the project. Requests to access these datasets should be directed to PhysioNet, https://physionet.org/, DOI: 10.13026/6mm1-ek67.

## Ethics statement

Ethical review and approval was not required for the study on human participants in accordance with the local legislation and institutional requirements. Written informed consent from the patients/participants or patients/participants' legal guardian/next of kin was not required to participate in this study in accordance with the national legislation and the institutional requirements.

## Author contributions

JJ: Data curation, Formal analysis, Writing – original draft. LY: Formal analysis, Writing – original draft. QZ: Formal analysis, Supervision, Writing – review & editing. MZ: Funding acquisition, Supervision, Writing – review & editing.
